# A two-phase digital tool to predict and prevent post-transplant hyperuricemia

**DOI:** 10.3389/fdgth.2026.1887452

**Published:** 2026-08-03

**Authors:** Min Wu, Limian Liang, Tian Tian, Chao Chen, Zongqiang Lai, Yunni Yu, Yourou Zheng, Wei Li, Shiyun Chen, Tian He, Wenmei Qiao, Qijun Chen, Zhenping Zhou, Miaona Liu, Haixia Sun

**Affiliations:** 1 Department of Pharmacy, Shenzhen Third People's Hospital, Shenzhen, Guangdong Province, China; 2 College of Pharmacy, Shenzhen Technology University, Shenzhen, Guangdong Province, China; 3 Department of Liver Transplantation, Shenzhen Third People's Hospital, Shenzhen, Guangdong Province, China; 4 Department of Kidney Transplantation, Shenzhen Third People's Hospital, Shenzhen, Guangdong Province, China

**Keywords:** clinical decision support, personalized medicine, post-transplant hyperuricemia, risk prediction, tacrolimus

## Abstract

**Background:**

Post-transplant hyperuricemia (PTHU) is a frequent and clinically significant metabolic complication among kidney transplant recipients. Current models lack personalized pre-transplant prevention strategies, and few studies define the optimal tacrolimus level for PTHU prevention. To address this unmet need, we developed and evaluated an integrated digital health tool consisting of a pre-operative risk-prediction algorithm and a post-operative tacrolimus dosing management module.

**Method:**

We enrolled 195 kidney transplant recipients. Pre-transplantation laboratory assessments and serial tacrolimus blood-concentration (Ctac) measurements longitudinally were conducted, while documenting adverse events. Logistic regression was used to identify the risk factors, and then incorporated into a nomogram. A subsequent dosing optimization algorithm was derived from these results.

**Results:**

We identified four independent risk factors—standard deviation of red blood cell distribution width (RDW-SD), prothrombin time activity (PTA), serum calcium, and Ctac. Notably, the model achieved strong discriminative performance, with AUROCs of 0.802 (95% CI 0.723−0.881, *p* < 0.001) pre-operatively and 0.824 (95% CI 0.734–0.913, *p* < 0.001) post-operatively. Using an optimal cutoff of 0.817, the dosing algorithm calculates an individualized maximum safe tacrolimus concentration for each patient.

**Conclusion:**

This study provides a pre-operative risk prediction model and a post-operative dosing tool that, used in tandem, can aid in the prevention of PTHU and the protection of renal allograft function.

## 1 Introduction

Kidney transplantation is the definitive treatment for end-stage renal disease (1). However, it necessitates lifelong immunosuppression for most recipients (2). These immunosuppressive regimens typically have a narrow therapeutic window, which can lead to severe complications such as cardiovascular disease, dyslipidemia, hyperglycemia, hyperuricemia, and life-threatening infections, all contributing to an increased risk of premature mortality (3, 4). Among these complications, post-transplant hyperuricemia (PTHU) is of particular concern, as multiple studies have demonstrated its independent association with graft failure and rejection (5–9).

Despite its clinical importance, evidence-based strategies for preventing PTHU remain limited (10, 11). Reported risk factors include elevated immunosuppressant trough levels (particularly tacrolimus), female gender, renal allograft dysfunction, increased serum creatinine, dyslipidemia, classical cardiovascular risk factors, diuretic use, and obesity (11–16). Some of these risk factors are not readily modifiable in the short term. Transplant recipients must maintain continuous immunosuppression (17), and intervening after transplantation is more complex due to potential drug-drug interactions. Therefore, preventive strategies initiated before transplantation are preferable. Moreover, although PTHU is a common complication of immunosuppressive therapy (18), few intelligent tools exist to guide individualized dose adjustments.

In summary, this study provides a dual strategy to mitigate PTHU. First, for pre-transplant patients, we developed a risk prediction model to identify high-risk individuals and facilitate early intervention for future transplant recipients. Second, for post-transplant patients, we introduced a tacrolimus dosing management tool to enable longitudinal monitoring and precise dose adjustment. Collectively, these measures are designed to jointly reduce the incidence of PTHU and improve long-term graft outcomes.

## 2 Materials and methods

### 2.1 Patient selection

This study included 195 kidney transplantation recipients admitted to Shenzhen Third People's hospital from January 1, 2023 to March 15, 2025. The enrolment criteria were as follows: (a) first-time kidney transplant recipients, (b) 18–70 years, (c) complete clinical data available, and (d) patients who were followed up regularly for more than 1 year. Exclusion criteria were age less than 18 years, patients with a history of hyperuricemia, combined any other organ transplantation, or patients lost to follow-up. This retrospective study was approved by Institutional Ethics Board of Shenzhen Third People's Hospital (Shenzhen, China) (2025-172-01) prior to data analysis. Our research conformed to the ethical standards of the Declaration of Helsinki. We certify that all organs were procured with written informed consent from the donor or the donor's legally authorized representative, without involvement of executed prisoners or any other source that violates international ethical standards.

### 2.2 Data collection

A comprehensive set of potential risk factors was evaluated, including demographic characteristics, baseline laboratory indices, pharmacogenetic data (CYP3A5 genotype), and key concomitant medications (e.g., Wuzhi Capsule) from the electronic medical record system. Adverse reactions and graft function were evaluated during follow-up.

### 2.3 Immunosuppressive regimen

All patients received a triple immunosuppressive regimen consisting of tacrolimus, mycophenolate mofetil (MMF), and corticosteroids. Immunosuppressive doses were adjusted based on therapeutic drug monitoring.

### 2.4 Tacrolimus therapeutic drug monitoring

Sample Preparation: Whole blood was collected by venipuncture into EDTA Vacutainer tubes. Whenever possible, trough samples were drawn just before the next scheduled dose to yield clinically relevant trough concentrations for dose optimization. Tubes were kept at 2–8 ℃ and analyzed within 12–24 h of collection to minimize pre-analytical variability.

Enzyme multiplied immunoassay technique (EMIT) assays were run on a Viva-ProE automated clinical chemistry analyzer (Siemens Healthcare Diagnostics). The platform operates under a rigorous quality-management system and monitors the reaction continuously at 340 nm with a high-precision spectrophotometric detection module.

The tacrolimus trough concentration (Ctac) was defined as the mean of all available measurements within the first three months post-transplantation. For patients diagnosed with post-transplant hyperuricemia (PTHU) during this period, only measurements taken strictly before the date of PTHU diagnosis were included in the calculation to avoid any temporal bias.

Tacrolimus intra-patient variability was assessed using the coefficient of variation, calculated according to the following formula: IPV (%) = (standard deviation/mean tacrolimus trough concentration) × 100 (19).

### 2.5 CYP3A5 genotyping

The patient's whole blood was processed using the separation kit (Shenzhou Medical Co., Ltd.) in accordance with the manufacturer's protocol for centrifugation (5 min, 5,000 rpm, 20 °C) to remove the white blood cell layer, which was then stored at −20 °C until the time of testing. At the time of testing, 5 μL of the stock solution was aspirated and added to the corresponding well, mixed, and then immediately run on the machine for detection (multi-channel fluorescence quantitative analyzer, Xi'an Tianlong Technology Co., Ltd.). Patients were classified by genotype into fast metabolizers (GG/GA) and slow metabolizers (AA), with slow metabolizers exhibiting higher tacrolimus blood concentrations and greater risk of drug accumulation.

### 2.6 Diagnosis of PTHU

According to the clinical diagnosis and treatment guidelines for hyperuricemia in Chinese kidney transplant recipients (2023) (20), the epidemiological definition of PTHU is as follows: under a normal purine diet, two separate fasting measurements of serum uric acid on non-consecutive days after transplantation exceed 420 μmol/L in male and postmenopausal female recipients, or 360 μmol/L in non-menopausal female recipients. The final diagnosis should be determined by the physician's comprehensive assessment. Recipients who met the diagnostic criteria for PTHU were stratified into two cohorts: the PTHU group and the non-PTHU group.

### 2.7 Determination of the optimal cut-off

The optimal probability cut-off value for the prediction model was determined by maximizing the Youden's index (J = sensitivity + specificity−1) derived from the receiver operating characteristic (ROC) curve. This method identifies the point on the ROC curve that optimizes the model's discriminative ability for clinical application.

### 2.8 Statistical analysis

Categorical variables were expressed as frequencies and percentages and compared using the *χ*^2^ test. Normally distributed continuous variables were expressed as mean ± standard deviation and compared using an independent t-test. Variables considered clinically relevant or showing a univariate association with PTHU (*p* < 0.1) were entered into a multivariate logistic regression model, with stepwise forward selection used to identify independent risk factors. Statistical significance for multivariate analyses was set at *p* < 0.05. Internal validation was performed using bootstrap resampling (500 replicates) to correct for over-optimism and assess model stability.

A nomogram was constructed based on the multivariate logistic regression model. The regression coefficients of the independent predictors were converted into corresponding point values, with the predictor showing the largest regression coefficient assigned 100 points. The total score was calculated by summing the points for each variable, and the estimated probability of PTHU was subsequently derived from the total score. Decision curve analysis was performed to evaluate the potential clinical utility of the nomogram.

Given the low proportion of missing data (<10% for all) and the assumption that data were missing at random, a complete-case analysis was employed for the logistic regression modeling. This approach excluded the small number of cases with any missing data from the final model fitting.

Statistical analyses were performed using SPSS version 26.0 (IBM, Armonk，NY), GraphPad Prism 8.0 and R software (ver 3.4.0, USA).

## 3 Results

### 3.1 Patient characteristics

A total of 242 kidney transplant recipients were initially enrolled in this study. After applying the exclusion criteria, 195 patients were included in the final analysis, of whom 142 (72.8%) developed PTHU. During follow-up, 95 (66.9%) patients developed PTHU in the first three months, 24 (16.9%) in the 4–6 months, and 23 (16.2%) in the 7–12 months (Table 1).

**Table 1:** Incidence of PTHU at different time periods post-transplantation.

Period (months)	1–3	4–6	7–12
PTHU cases (n, %)	95 (66.9)	24 (16.9)	23 (16.2)

PTHU, post-transplant hyperuricemia.

Among the 195 patients, 123 (63.0%) were male. There were 142 and 53 patients in the PTHU and non-PTHU group, respectively. The clinical characteristics of the two groups were shown in Table 2 and Supplementary Table S1. The mean tacrolimus blood concentration (C_tac_, μg/mL) during the first three months post-transplantation was significantly higher in the PTHU group compared to the non-PTHU group (8.89 vs. 8.10, *p* *<* 0.001). Patients in the PTHU group had a higher serum calcium concentration (Ca, mmol/L) than those in the non-PTHU group (2.15 vs. 1.98, *p* *<* 0.001). The standard deviation of red blood cell distribution width (RDW-SD, fL) in PTHU group was significantly lower than that in the non-PTHU group (45.04 vs. 48.13, *p* *<* 0.001). Patients in the PTHU group had a lower prothrombin time activity (PTA, %) than that in the non-PTHU group (100.92 vs. 109.53, *p* *<* 0.001). Furthermore, no significant differences were observed between the two groups in many other characteristics, such as pre-transplant serum uric acid levels, age, liver function, concomitant medications, among others.

**Table 2:** Characteristics of participants in PTHU group and non-PTHU group.

Indicator	PTHU (*n* = 142)	Non-PTHU (*n* = 53)	t/*χ*^2^-value	*p*-value
The Blood Concentration of Tacrolimus				
C_tac_ (*μ*g/mL)	8.89 ± 1.45	8.10 ± 1.08	3.854	<0.001
Complete Blood Count				
RDW-SD (fL)	45.04 ± 3.86	48.13 ± 4.52	­4.491	<0.001
Coagulation Profile				
PTA (%)	100.92 ± 10.15	109.53 ± 11.21	-4.783	<0.001
Renal Function & Biochemistry				
Ca (mmol/L)	2.15 ± 0.36	1.98 ± 0.15	4.022	<0.001

Continuous data are presented as mean ± standard deviation; categorical data are presented as number (percentage). *p* < 0.05 indicates that the difference is statistically significant. RDW-SD, red blood cell distribution width-standard deviation; PTA, prothrombin time activity; Ca, serum calcium concentration; C_tac_,The blood concentration of tacrolimus.

### 3.2 Tacrolimus concentration and intra-patient variability status

**Figure 1:**
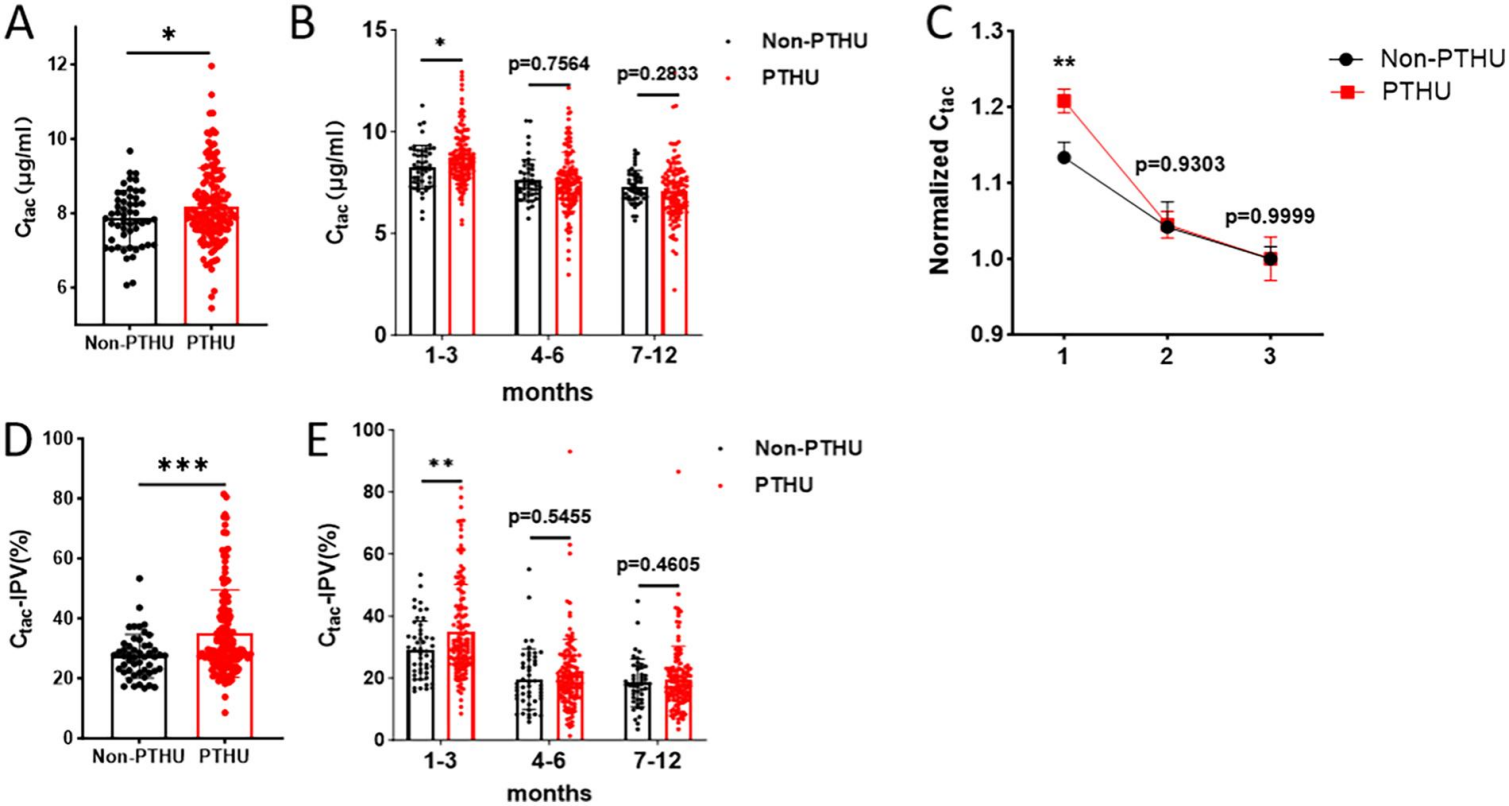
Tacrolimus concentration and intra-patient variability status. **(A)** Mean tacrolimus concentration during the first post-transplant year in the PTHU and non-PTHU groups. **(B)** Mean tacrolimus concentration at 1–3, 4–6, and 7–12 months post-transplant in the PTHU and non-PTHU groups. **(C)** Tacrolimus concentrations at 1–3 and 4–6 months normalized to the 7–12-month value: 1 = (1–3 months)/(7–12 months); 2 = (4–6 months)/(7–12 months); 3 = (7–12 months)/(7–12 months). **(D)** Intra-patient variability over the first post-transplant year in the PTHU and non-PTHU groups. **(E)** Intra-patient variability at 1–3, 4–6, and 7–12 months post-transplant in the PTHU and non-PTHU groups. *p<0.05, **p<0.01, ***p<0.001.

During the first year post-transplant, tacrolimus concentration was significantly higher in the PTHU group than in the non-PTHU group (Figure 1A), particularly within the first three months (Figure 1B). When the blood drug concentration at the transplantation stable stage was taken as the baseline, the PTHU group exhibited a greater change in concentration over the first three months (Figure 1C). Analysis of tacrolimus intra-patient variability showed that the PTHU group had higher variability (Figure 1D), especially during the first three months (Figure 1E).

### 3.3 Risk factors of PTHU

Univariate logistic regression analysis identified several potential risk factors for PTHU with a *p-value* less than 0.10, including C_tac_, Ca, RDW-SD, PTA, body weight before transplantation, myoglobin, serum sodium concentration, and CYP3A5 genotype. These variables were subsequently analyzed using multivariate logistic regression to identify independent predictors of PTHU. The final model revealed that only four factors—C_tac_, Ca, RDW-SD and PTA—were independently associated with the development of PTHU (*p* < 0.05) (Table 3). The potential confounders, including body weight before transplantation, myoglobin, serum sodium concentration, and CYP3A5 genotype, did not significantly affect the final model.

**Table 3:** Univariate and multivariate logistic regression analyses in participants for PTHU risk prediction.

Variables	Univariate analysis		Multivariate analysis	
	OR (95% CI)	*p*_1_-value	OR (95% CI)	*p*_2_-value
C_tac_ (μg/mL)	1.736 (1.229–2.454)	0.002	1.586 (1.058–2.377)	0.026
Pre-op Weight (kg)	1.026 (0.996–1.056)	0.087	NA	
RDW-SD (fL)	0.934 (0.874–0.997)	0.042	0.840 (0.752–0.937)	0.002
Mb (ng/mL)	1.003 (1.000–1.006)	0.095	NA	
PTA (%)	0.975 (0.949–1.001)	0.060	0.928 (0.882–0.977)	0.004
Na (mmol/L)	1.100 (0.998–1.211)	0.054	NA	
Ca (mmol/L)	4.369 (1.049–18.188)	0.043	42.41 (3.366–534.372)	0.004
CYP3A5	1.670 (1.013–2.754)	0.044	NA	

*p*_1_ < 0.10 was considered statistically significant in univariate logistic regression*,* whereas *p*_2_ < 0.05 was considered statistically significant in multivariate logistic regression.

To assess multicollinearity, collinearity diagnostics were conducted on the identified independent risk factors, and all tolerance values were found to be below 0.5 (Supplementary Table S2), suggesting that there was no significant multicollinearity.

### 3.4 Risk prediction models of PTHU

**Figure 2:**
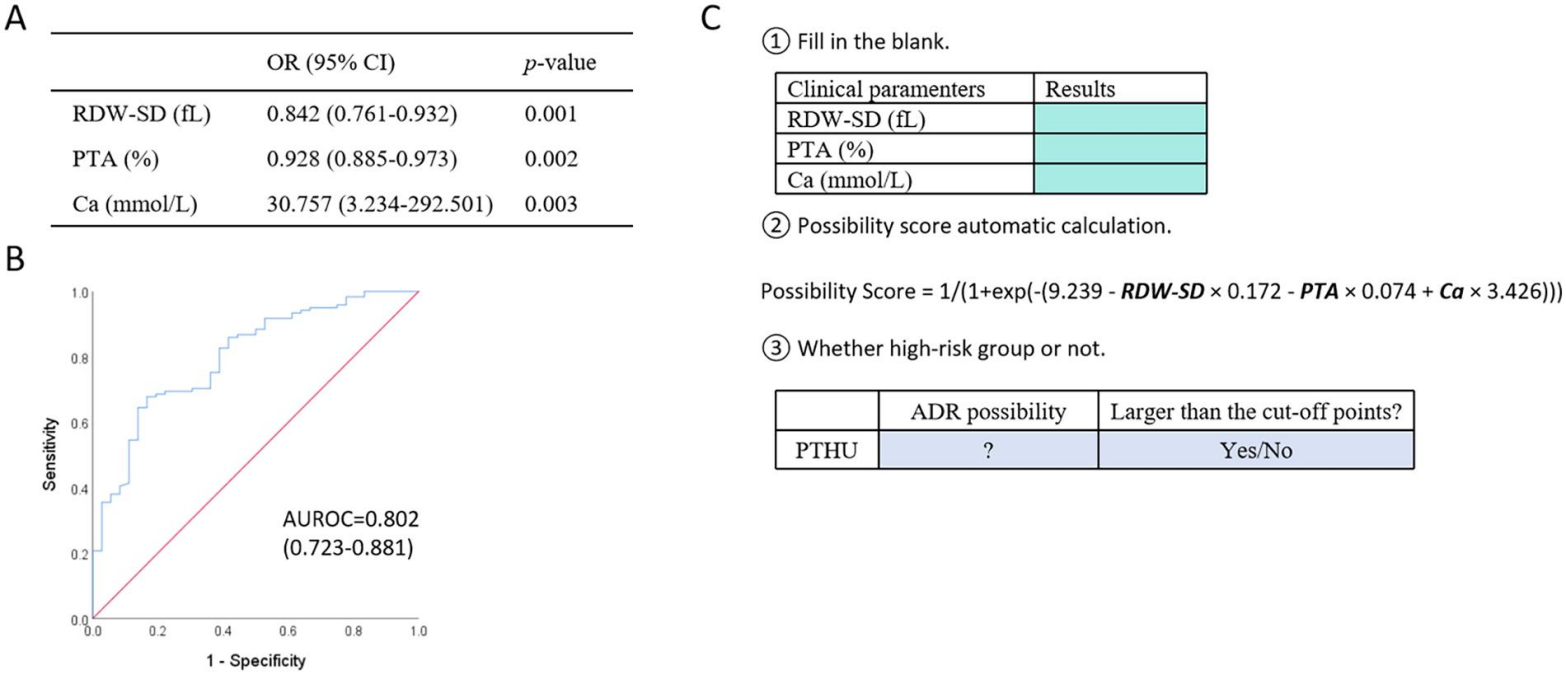
Risk prediction models for PTHU prior to kidney transplantation. **(A)** Multivariate logistic regression analysis. **(B)** ROC curve of the development PTHU risk prediction model. **(C)** Corresponding scoring system.

We developed a predictive model to identify high-risk populations for PTHU prior to kidney transplantation. Based on the multivariate logistic regression analysis（Table 3), three independent risk factors were incorporated into the model (Figure 2A), and the receiver operating characteristic (ROC) curve is shown in Figure 2B. The risk prediction model included Ca, RDW-SD, and PTA. The area under the receiver operating characteristic curve (AUROC) was 0.802 (95% CI, 0.720–0.881; *p* < 0.001), indicating excellent discriminative ability. The optimal cut-off value of the predictive score was 0.827, with a sensitivity of 67.8% and a specificity of 83.3%. A corresponding scoring system was developed (Figure 2C). By entering the values of each risk factor, the PTHU risk score can be automatically calculated, and cases exceeding the cut-off value are identified as high risk for developing PTHU.

### 3.5 Tacrolimus dosing management tool

**Figure 3:**
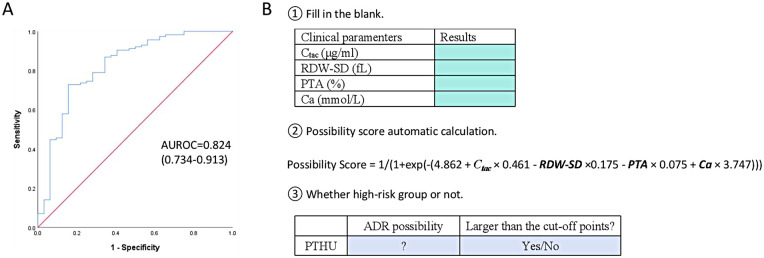
Risk prediction models for PTHU after transplantation. **(A)** ROC curve of the development PTHU risk prediction model. **(B)** Corresponding scoring system.

For post-transplantation patients, we build a tacrolimus dosing management tool to reduce the risk of PTHU. Based on the logistic multivariate regression analysis, the four independent risk factors were included in the prediction model after transplantation (Table 3), and the ROC curve was shown in Figure 3A. Risk prediction model include C_tac_, Ca, RDW-SD, and PTA, and the AUROC was 0.824 (95% CI 0.734–0.913, *p* < 0.001), suggesting that the prediction model has an excellent discrimination. Using the Youden's index as described in the Methods, the optimal cut-off value for the prediction score was identified as 0.817. The sensitivity was 72.8%. The specificity was 84.4%. According to the cut-off values, the maximum safe tacrolimus concentration for each individual after transplantation was calculated as following:

$${\text{C}}_{\text{tac}}\text{ = }\frac{\text{ - ln(1/ 0}\text{.824 - 1) - 4}.862+\mathrm{RDW}-\mathrm{SD}\times 0.175+\text{PTA}\times 0.075-Ca\times 3.747}{0.461}$$

Furthermore, we created a tacrolimus dosing management tool to facilitate long-term monitoring of post-transplantation patients accordingly (Figure 3B). When you fill in the values for each risk factors, the PTHU risk score is calculated automatically. When the risk scores exceed the cut-off values, it indicate a high risk of occurring PTHU. Combined with other physical condition, we can adjust tacrolimus concentration individually.

A sensitivity analysis was performed by forcing CYP3A5 genotype into the multivariate model. The pre-operative model yielded an AUROC of 0.818, and the post-operative model yielded an AUROC of 0.836 (Supplementary Figure S1), indicating no meaningful improvement over the original model (AUROC 0.802 and 0.824, respectively).

### 3.6 Nomogram development

**Figure 4:**
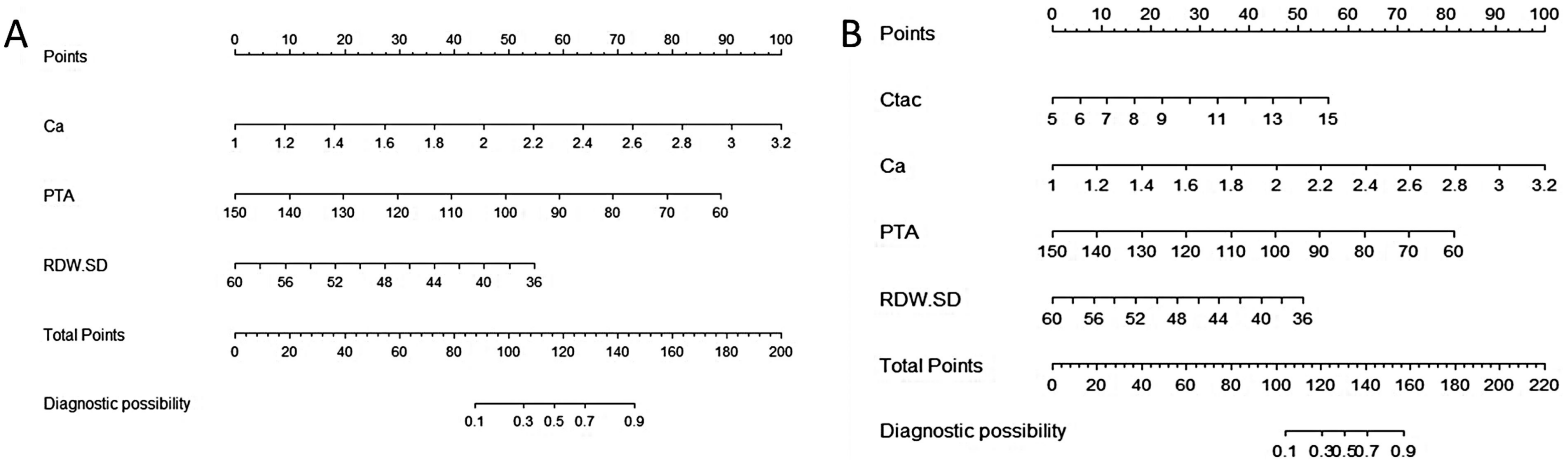
Nomogram evaluation charts. **(A)** Before surgery. **(B)** During long-term medication after surgery.

Based on the above analysis, nomograms were established for pre- and post-transplant risk assessment of PTHU (Figures 4A,B).

### 3.7 Nomogram validation

**Figure 5:**
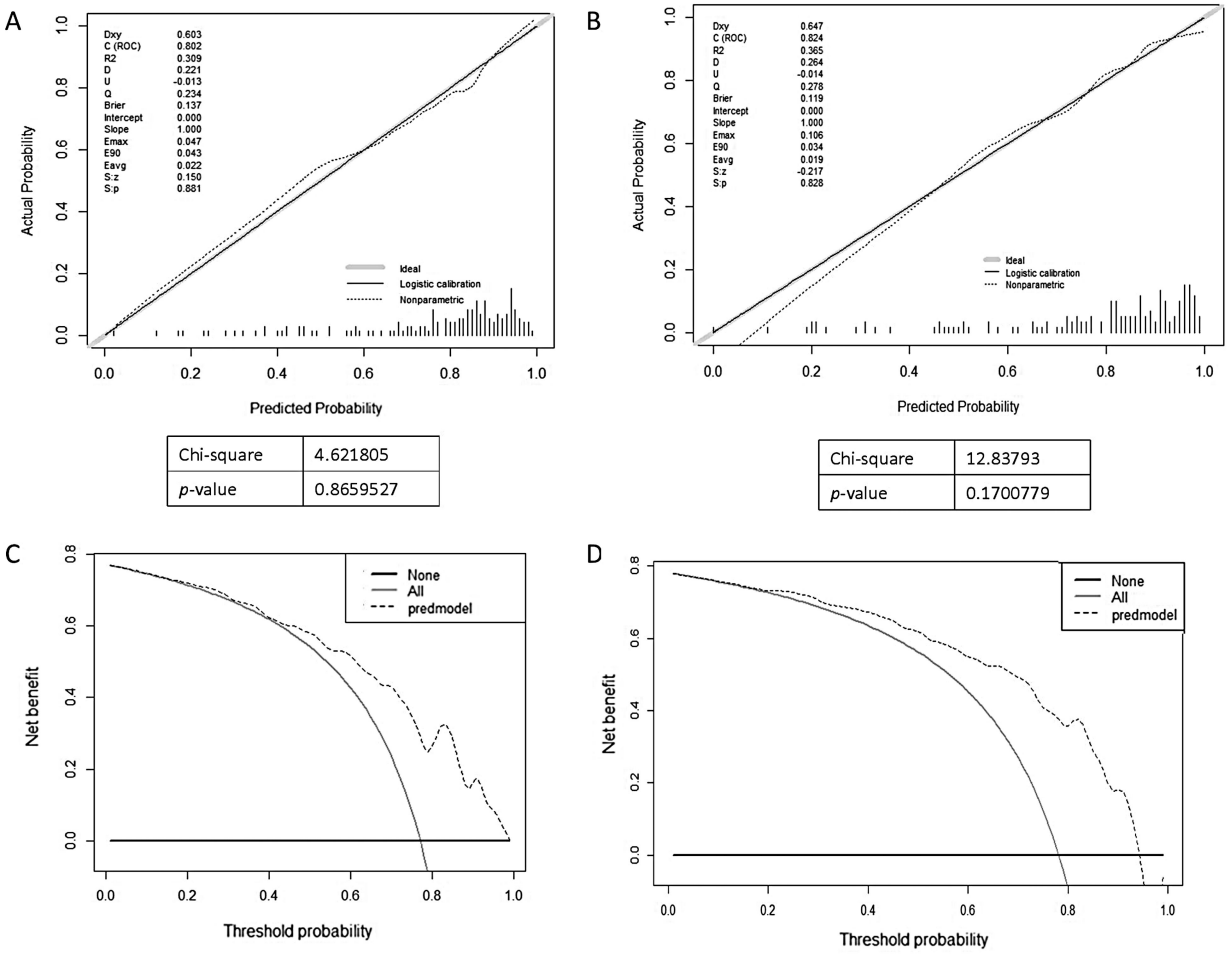
Nomogram validation. **(A)** Calibration plots for patients before surgery. **(B)** Calibration plots for patients after surgery. **(C)** Decision curve analysis for patients before surgery. **(D)** Decision curve analysis for patients after surgery.

To evaluate the clinical utility of the predictive model, we utilized calibration plots. As can be observed, the model exhibited good agreement between the estimated and observed risks (Figures 5A,B). Furthermore, the *p*-value exceeds 0.01 in both calibration plots.

Next, we conducted decision curve analysis (DCA), and multi-index to predict the correct prediction of PTHU (Figures 5C,D). The net benefit of the multi-index model exhibited a wider threshold probability range.

### 3.8 Internal validation

**Figure 6:**
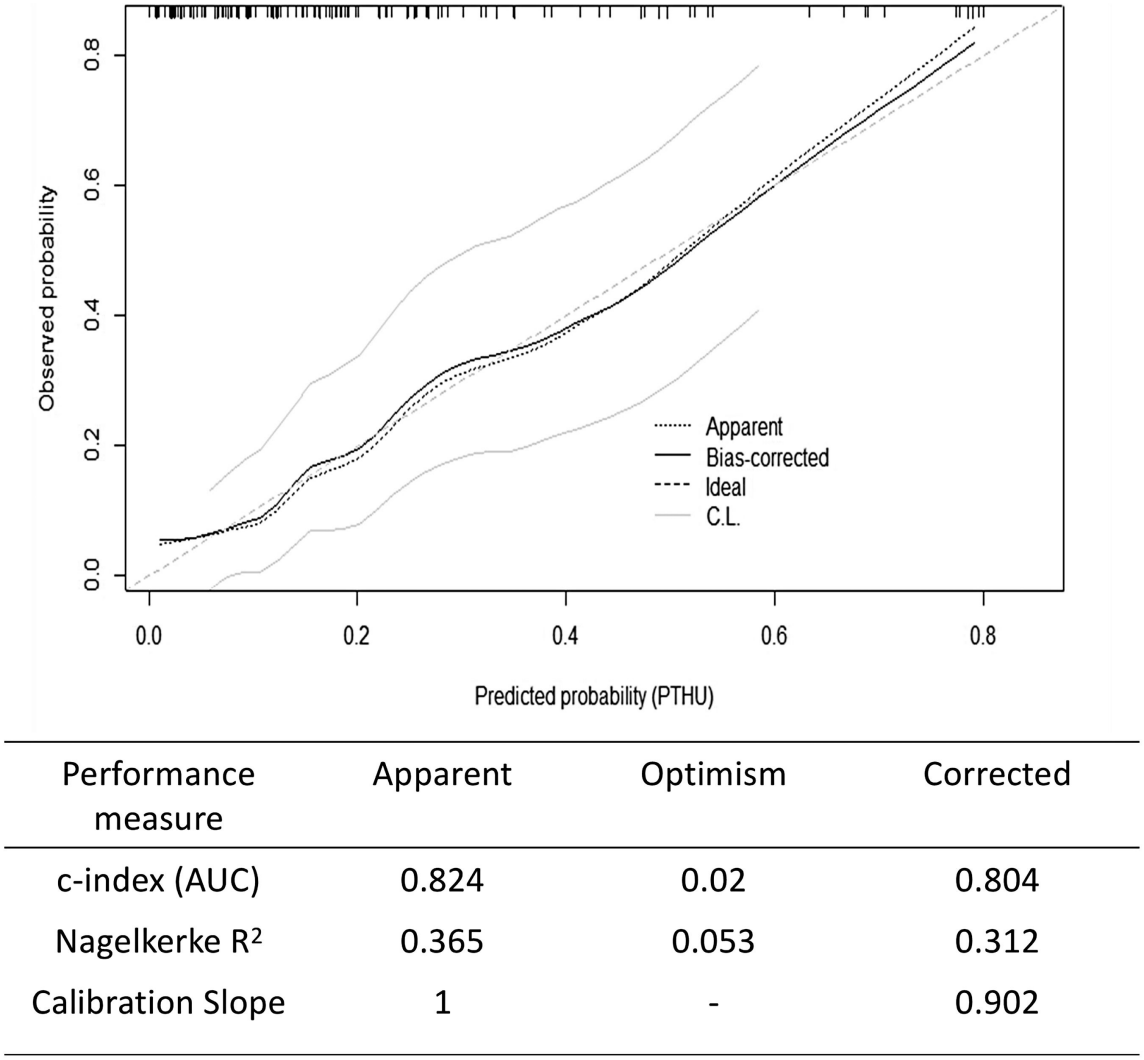
Bootstrap-calibrated calibration curve of the diagnostic model. Calibration was assessed using 500 bootstrap resamples to correct for optimism.

Bootstrap optimism correction (B = 500) revealed a small optimism of 0.020, resulting in an optimism-corrected C-index of 0.804. The Nagelkerke R^2^ was 0.365 (apparent) vs. 0.312 (corrected). The calibration slope decreased from 1.000 (apparent) to 0.902 after correction, suggesting only mild over-optimism. The bootstrap-corrected calibration curve (Figure 6) showed excellent agreement between predicted and observed probabilities, with a mean absolute error of 0.02. These results indicate that the model is internally valid and not substantially affected by overfitting.

## 4 Discussion

PTHU is a risk factor for early allograft dysfunction (21, 22); however, personalized strategies for its management remain poorly defined (10, 11). Managing PTHU after transplantation is particularly challenging because patients require ongoing immunosuppression. To protect the allograft, it is crucial to minimize the risk of drug-drug interactions, which complicates and often delays effective intervention. In contrast, intervention before transplantation is logistically simpler and allows more time for implementation. Therefore, our study established a pre-transplant prediction model to identify high-risk patients. For those already transplanted, we also built a tacrolimus dosing management tool to guide individualized therapy.

It is well-established that anemia is common in patients with chronic kidney disease (CKD), with hemoglobin levels serving as a key parameter for assessment and management (23). While several pharmacological agents aim to increase hemoglobin, few directly target the red cell distribution width-standard deviation (RDW-SD), an often-overlooked parameter. Consistent with our findings, hemoglobin levels did not differ significantly between PTHU and non-PTHU patients, whereas RDW-SD did. In clinical practice, different hemoglobin-raising medications operate via distinct mechanisms—some primarily promoting red blood cell maturation, others enhancing erythrocyte production (24). This underscores the need for a more rational, combination therapeutic strategy. The goal should not only be to achieve target hemoglobin levels but also to favorably modulate RDW-SD, potentially leading to more comprehensive hematological and clinical outcomes.

Abnormal calcium and phosphorus metabolism is common in CKD patients (25). Higher serum calcium levels are associated with an increased risk of both hyperuricemia (26) and vascular calcification (27). In CKD, damage to renal units impairs the kidney's ability to synthesize active vitamin D (28), often necessitating supplementation to prevent hypocalcemia. However, after kidney transplantation, as renal function recovers, patients become susceptible to hypercalcemia—a condition detrimental to the allograft. Therefore, maintaining lower serum calcium levels before-transplantation may help prevent problematic hypercalcemia after-transplantation.

For post-transplantation patients, we developed a tacrolimus dosing management tool. Consistent with previous reports (29), we observed that most of PTHU cases occurred within the first three months after transplantation. Tacrolimus is a cornerstone immunosuppressive regimen for protecting the renal allograft (30). Currently, tacrolimus dosing is largely adjusted based on clinical experience. Without intelligent tools, it is difficult to minimize adverse drug reactions while maintaining optimal graft protection. Consequently, hyperuricemia commonly occurs. Importantly, our data revealed that patients who developed PTHU exhibited higher intra-patient variability (IPV) in tacrolimus concentrations compared to those without PTHU. This finding highlights the clinical need to minimize IPV and maintain stable drug exposure. Our tool provides an upper concentration threshold to avoid PTHU; when combined with the standard anti-rejection therapeutic window, clinicians can aim to keep the patient's tacrolimus levels within the overlapping safe zone for the majority of the time. For patients with high IPV, more frequent therapeutic drug monitoring (TDM) is warranted to achieve this goal. A personalized tacrolimus dosing tool is essential to balance the risks of immunological rejection and drug-related adverse effects. Future iterations of this tool could integrate IPV metrics and dynamic TDM data to enable real-time dose adjustments, further enhancing its clinical utility.

Limitations of this study should be acknowledged. First, the single-center retrospective design with a relatively small sample size may limit the generalizability of our findings. The observed high incidence of PTHU (72.8%) in our cohort may partly reflect regional dietary habits that predispose to hyperuricemia, which could introduce selection bias and affect the composition of the study population. Consequently, the proposed nomogram and dosing tool require external validation in multicenter, prospective cohorts to confirm their efficacy and clinical utility before widespread implementation. Second, although selection bias cannot be entirely excluded, strict inclusion criteria and standardized clinical sampling were employed to enhance the real-world representativeness of our cohort. Most importantly, the current dosing tool primarily focuses on mitigating hyperuricemia risk. In clinical practice, tacrolimus dosing must balance the prevention of allograft rejection with the management of metabolic side effects. Our tool is designed as a clinical decision support system, not a replacement for clinical judgment. It provides an upper safety limit for tacrolimus to minimize PTHU risk. If the calculated “maximum safe concentration” falls below the lower limit of the therapeutic window required for rejection prophylaxis, priority should be given to maintaining anti-rejection efficacy, with enhanced uric acid monitoring and management. Future iterations of this tool should integrate rejection risk metrics to optimize this balance more comprehensively.

In conclusion, this study developed and validated a personalized nomogram to predict the risk of PTHU in pre-transplantation patients. This tool enables accurate risk stratification, facilitating tailored management before transplantation. Furthermore, for post-transplantation, we established a tacrolimus dosing management tool to mitigate PTHU. By integrating pre-transplant metabolic profiling with post-transplant drug monitoring, our work establishes an integrated, two-phase management paradigm. This strategy allows for proactive intervention before transplantation and continuous optimization afterward, with the ultimate goal of minimizing PTHU occurrence and protecting renal allograft function.

## Data Availability

The datasets presented in this study can be found in online repositories. The names of the repository/repositories and accession number(s) can be found in the article/Supplementary Material.

## Glossary

ALB: albumin
ALT: alanine aminotransferase
APTT: activated partial thromboplastin time
APTT-R: APTT ratio
AST: aspartate aminotransferase
AT-III: antithrombin III
AUROC: Area under the receiver operating characteristic curve
BA#: basophil absolute count
BA%: basophil percentage
BMI: body mass index
BUN: blood urea nitrogen
CAI: chronic allograft injury
Ca: serum calcium concentration
Ccr: creatinine clearance rate
CK: creatine kinase
CK-MB: creatine kinase-MB
Cl: chloride
Cr: creatinine
Ctac: tacrolimus blood concentration
CsA: cyclosporine A
CYP3A5: cytochrome P450 3A5
D-D: D-dimer
DBIL: direct bilirubin
DCA: decision curve analysis
DNA: deoxyribonucleic acid
EDTA: ethylenediaminetetraacetic Acid
EMIT: enzyme multiplied immunoassay technique
EO#: eosinophil absolute count
EO%: eosinophil percentage
FBG: fasting blood glucose
FIB: fibrinogen
FIB-s: fibrinogen-seconds
GLOB: globulin
HCO₃⁻: bicarbonate
HCT: hematocrit
HGB: hemoglobin
INR: international normalized ratio
K: potassium
KM: Kaplan–Meier
LDH: lactate dehydrogenase
LY#: lymphocyte absolute count
LY%: lymphocyte percentage
MCH: mean corpuscular hemoglobin
MCHC: mean corpuscular hemoglobin concentration
MCV: mean corpuscular volume
Mb: myoglobin
MMF: mycophenolate mofetil
MO#: monocyte absolute count
MO%: monocyte percentage
MPV: mean platelet volume
Na: sodium
NE#: neutrophil absolute count
NE%: neutrophil percentage
NT-proBNP: N-terminal pro-B-type natriuretic peptide
P: phosphorus
PCT: plateletcrit
PDW: platelet distribution width
PLT: platelet count
P-LCR: large platelet ratio
PRE-op Cr: preoperative last creatinine
Pre-op Height: preoperative height
Pre-op Weight: preoperative weight
PT: prothrombin time
PTA: prothrombin time activity
PT-R: prothrombin time ratio
PTHU: post-transplant hyperuricemia
RBC: red blood cell count
RDW-CV: red cell distribution width-coefficient of variation
RDW-SD: Red cell distribution width-standard deviation
ROC: receiver operating characteristic
TDM: therapeutic drug monitoring
TBIL: total bilirubin
TC: total cholesterol
Tg: triglycerides
TP: total protein
TnI: troponin I
TT: thrombin time
UA: uric acid
WBC: white blood cell count
CKD: chronic kidney disease.
