# Supplementary material for: A two-phase digital tool to predict and prevent post-transplant hyperuricemia
Source: Front Digit Health. 2026 Aug 3;8:1887452. doi: 10.3389/fdgth.2026.1887452 (PMC13478219; doi:10.3389/fdgth.2026.1887452)
Supplement: Supplementary file 1 [file Table1.docx]

A Two-Phase Digital Tool to Predict and Prevent Post-Transplant Hyperuricemia

**Min Wu^1,†^, Limian Liang^1,†^, Tian Tian^2,†^, Chao Chen^1^, Zongqiang Lai^1^, Yunni Yu^1^, Yourou Zheng^1^, Wei Li^1^, Shiyun Chen^1^, Tian He^1^, Wenmei Qiao^1^, Qijun Chen^3^, Zhenping Zhou^4^, Miaona Liu^1,*^, Haixia Sun^1,*^**

^1^ Department of Pharmacy, Shenzhen Third People’s Hospital, Shenzhen, Guangdong Province 518112, China.

^2^ College of Pharmacy, Shenzhen Technology University, Shenzhen, Guangdong Province 518118, China.

^3^ Department of Liver Transplantation, Shenzhen Third People’s Hospital, Shenzhen, Guangdong Province 518112, China.

^4^ Department of Kidney Transplantation, Shenzhen Third People’s Hospital, Shenzhen, Guangdong Province 518112, China.

**^†^Joint first authorship**

**^*^ Corresponding :**

Haixia Sun

*E-mail address*: sunhx7@alumni.sysu.edu.cn

Table S1. Characteristics of participants in PTHU group and non-PTHU group.

| Indicator | | PTHU (n=142) | Non-PTHU (n=53) | t/χ²-value | *p*-value |
| --- | --- | --- | --- | --- | --- |
| Demographics & Anthropometrics | | | | | |
| Age (years) | | 41.39 ± 10.56 | 42.45 ± 10.42 | -0.625 | 0.533 |
| Male(n,%) | | 94(66.20) | 29(54.72) | 2.184 | 0.139 |
| Pre-op Weight (kg) | | 60.88 ± 11.41 | 57.78 ± 10.31 | 1.729 | 0.085 |
| Pre-op Height (cm) | | 166.42 ± 8.11 | 164.94 ± 8.86 | 1.099 | 0.273 |
| BMI (kg/m²) | | 21.89 ± 3.36 | 21.18 ± 3.16 | 1.322 | 0.188 |
| Drug Combination | | | | | |
| Wuzhi Capsule (n,%) | | 58(40.85) | 28(52.83) | 2.249 | 0.134 |
| Tacrolimus Blood Concentration | | | | | |
| C_tac_ (μg/ml) | | 8.89 ± 1.45 | 8.10 ± 1.08 | 3.854 | **<0.001** |
| CYP3A5 Genotype | | | | | |
| GG (n,%) | | 67 (55.83) | 18 (39.13) | 4.182 | 0.124 |
| GA (n,%) | | 43 (35.83) | 21 (45.65) |  |  |
| AA (n,%) | | 10 (8.33) | 7 (15.22) |  |  |
| Complete Blood Count | | | | | |
| WBC (10⁹/L) | | 6.54 ± 1.86 | 6.90 ± 2.07 | -1.182 | 0.239 |
| P-LCR (%) | | 22.73 ± 6.68 | 22.70 ± 7.49 | 0.032 | 0.974 |
| MO% (%) | | 8.55 ± 2.49 | 8.40 ± 1.88 | 0.391 | 0.696 |
| MO# (10⁹/L) | | 0.54 ± 0.18 | 0.58 ± 0.21 | -1.035 | 0.302 |
| RDW-CV (%) | | 13.86 ± 1.61 | 14.10 ± 1.81 | -0.872 | 0.384 |
| RDW-SD (fL) | | 45.04 ± 3.86 | 48.13 ± 4.52 | -4.491 | **<0.001** |
| RBC (10¹²/L) | | 3.98 ± 0.71 | 3.86 ± 0.67 | 1.070 | 0.286 |
| HCT (%) | | 36.10 ± 5.67 | 35.62 ± 6.46 | 0.510 | 0.611 |
| LY% (%) | | 22.60 ± 7.14 | 21.17 ± 7.16 | 1.246 | 0.214 |
| LY# (10⁹/L) | | 1.43 ± 0.49 | 1.43 ± 0.58 | 0.030 | 0.976 |
| MCV (fL) | | 91.23 ± 6.80 | 92.53 ± 8.41 | -1.111 | 0.268 |
| MCH (pg) | | 29.79 ± 2.77 | 30.17 ± 3.64 | -0.783 | 0.435 |
| MCHC (g/L) | | 326.14 ± 13.17 | 325.19 ± 16.54 | 0.418 | 0.677 |
| MPV (fL) | | 9.76 ± 0.86 | 9.73 ± 0.96 | 0.237 | 0.813 |
| BA# (10⁹/L) | | 0.04 ± 0.02 | 0.04 ± 0.02 | 0.390 | 0.697 |
| BA% (%) | | 0.64 ± 0.32 | 0.57 ± 0.28 | 1.489 | 0.138 |
| EO% (%) | | 3.60 ± 2.49 | 3.43 ± 2.46 | 0.442 | 0.659 |
| EO# (10⁹/L) | | 0.23 ± 0.18 | 0.23 ± 0.17 | 0.129 | 0.897 |
| HGB (g/L) | | 117.63 ± 18.39 | 115.75 ± 21.28 | 0.605 | 0.546 |
| PDW (%) | | 10.54 ± 1.72 | 10.46 ± 1.78 | 0.298 | 0.766 |
| PLT (10⁹/L) | | 208.11 ± 60.20 | 199.32 ± 57.44 | 0.918 | 0.360 |
| PCT (%) | | 0.20 ± 0.05 | 0.20 ± 0.05 | 0.933 | 0.352 |
| NE% (%) | | 64.61 ± 9.03 | 66.44 ± 8.33 | -1.285 | 0.200 |
| NE# (10⁹/L) | | 4.29 ± 1.57 | 4.63 ± 1.66 | -1.346 | 0.180 |
| Emergency Biochemistry | | | | | |
| Na (mmol/L) | | 138.74 ± 2.99 | 138.01 ± 3.66 | 1.421 | 0.157 |
| K (mmol/L) | | 4.28 ± 0.74 | 4.34 ± 0.96 | -0.473 | 0.637 |
| Cl (mmol/L) | | 98.91 ± 3.47 | 98.28 ± 3.95 | 1.082 | 0.280 |
| CO₂ (mmol/L) | | 25.12 ± 4.23 | 26.12 ± 5.64 | -1.330 | 0.185 |
| Cr (umol/L) | | 1038.21 ± 297.74 | 972.83 ± 292.63 | 1.370 | 0.172 |
| Urea (mmol/L) | | 19.33 ± 6.32 | 20.33 ± 7.66 | -0.933 | 0.352 |
| Cardiac Biochemistry | | | | | |
| NT-proBNP (pg/mL) | 3244.23 ± 4604.04 | | 4077.18 ± 5871.52 | -1.023 | 0.308 |
| TnI (ug/L) | | 0.015 ± 0.009 | 0.018 ± 0.015 | -1.452 | 0.148 |
| Mb (ng/mL) | | 274.57 ± 241.45 | 226.84 ± 103.78 | 1.390 | 0.166 |
| CK (U/L) | | 128.75 ± 287.21 | 93.42 ± 57.58 | 0.879 | 0.380 |
| CK-MB (ng/mL) | | 0.73 ± 0.89 | 0.76 ± 1.08 | -0.181 | 0.856 |
| LDH (U/L) | | 185.13 ± 42.61 | 183.30 ± 40.04 | 0.270 | 0.787 |
| Coagulation Profile | | | | | |
| APTT Ref (s) | | 34.00 ± 0.00 | 34.00 ± 0.00 | - | - |
| D-D (ug/mL) | | 0.44 ± 0.43 | 0.49 ± 0.52 | -0.694 | 0.488 |
| TT Ratio | | 0.87 ± 0.06 | 0.87 ± 0.06 | -0.239 | 0.812 |
| TT Ref (s) | | 21.00 ± 0.00 | 21.00 ± 0.00 | - | - |
| APTT (s) | | 36.05 ± 4.11 | 35.50 ± 3.36 | 0.878 | 0.381 |
| APTT-R | | 1.06 ± 0.12 | 1.04 ± 0.10 | 0.869 | 0.386 |
| AT-III (%) | | 93.51 ± 14.33 | 93.50 ± 14.52 | 0.003 | 0.998 |
| INR | | 0.99 ± 0.07 | 0.97 ± 0.06 | 1.828 | 0.069 |
| TT (s) | | 18.26 ± 1.19 | 18.31 ± 1.25 | -0.284 | 0.777 |
| PT-R | | 0.99 ± 0.05 | 0.98 ± 0.05 | 1.815 | 0.071 |
| PTA (%) | | 100.92 ± 10.15 | 109.53 ± 11.21 | -4.783 | **<0.001** |
| FIB (g/L) | | 3.69 ± 0.99 | 3.71 ± 0.96 | -0.115 | 0.908 |
| FIB-s (s) | | 14.10 ± 3.35 | 14.03 ± 3.37 | 0.147 | 0.883 |
| PT (s) | | 13.18 ± 0.73 | 13.02 ± 0.70 | 1.417 | 0.158 |
| Renal Function & Biochemistry | | | | | |
| Pre-op Cr (umol/L) | | 1047.12 ± 299.53 | 991.12 ± 300.35 | 1.161 | 0.247 |
| Ccr (mL/min•1.73m²) | | 6.90 ± 2.15 | 6.87 ± 2.81 | 0.069 | 0.945 |
| BUN (mmol/L) | | 19.61 ± 6.41 | 21.10 ± 8.01 | -1.344 | 0.180 |
| UA (umol/L) | | 367.73 ± 89.89 | 376.20 ± 80.84 | -0.600 | 0.549 |
| ALT (IU/L) | | 17.49 ± 10.49 | 17.97 ± 12.99 | -0.266 | 0.791 |
| AST (IU/L) | | 22.05 ± 9.14 | 23.01 ± 8.16 | -0.669 | 0.504 |
| TBIL (umol/L) | | 9.19 ± 4.52 | 8.65 ± 3.47 | 0.778 | 0.437 |
| DBIL (umol/L) | | 3.54 ± 2.91 | 3.05 ± 2.38 | 0.855 | 0.394 |
| ALB (g/L) | | 43.86 ± 4.74 | 42.61 ± 5.22 | 1.600 | 0.111 |
| GLOB (g/L) | 32.69 ± 6.63 | | 34.69 ± 16.25 | -1.228 | 0.221 |
| TP (g/L) | 76.47 ± 9.70 | | 77.32 ± 18.63 | -0.415 | 0.679 |
| TC (mmol/L) | 3.95 ± 0.95 | | 3.81 ± 0.84 | 0.749 | 0.456 |
| TG (mmol/L) | 1.66 ± 1.04 | | 1.43 ± 0.71 | 1.170 | 0.244 |
| Na (mmol/L) | 138.67 ± 2.98 | | 137.64 ± 3.84 | 1.961 | 0.051 |
| Ca (mmol/L) | 2.15 ± 0.36 | | 1.98 ± 0.15 | 4.022 | **<0.001** |
| K (mmol/L) | 4.28 ± 0.77 | | 4.41 ± 0.99 | -0.929 | 0.354 |
| P (mmol/L) | 1.50 ± 0.60 | | 1.47 ± 0.45 | 0.279 | 0.780 |
| HCO₃⁻ (mmol/L) | 25.08 ± 4.37 | | 26.16 ± 5.84 | -1.391 | 0.166 |
| FBG (mmol/L) | 9.56 ± 44.38 | | 6.14 ± 2.14 | 0.560 | 0.576 |

Note: Continuous data are presented as mean ± standard deviation; categorical data are presented as number ( percentage). *p* < 0.05 indicates that the difference is statistically significant. PTHU, post-transplant hyperuricemia.Abbreviations: ALB, Albumin; ALT, Alanine Aminotransferase; APTT, Activated Partial Thromboplastin Time; APTT-R, APTT Ratio; AST, Aspartate Aminotransferase; AT-III, Antithrombin III; BA%, Basophil Percentage; BA#, Basophil Absolute Count; BMI, Body Mass Index; BUN, Blood Urea Nitrogen; Ca, serum calcium concentration; Ccr, Creatinine Clearance Rate; CK, Creatine Kinase; CK-MB, Creatine Kinase-MB; Cl, Chloride; Cr, Creatinine; C_tac_,The blood concentration of tacrolimus; CYP3A5, Cytochrome P450 3A5; D-D, D-Dimer; DBIL, Direct Bilirubin; EO%, Eosinophil Percentage; EO#, Eosinophil Absolute Count; FBG, Fasting Blood Glucose; FIB, Fibrinogen; FIB-s, Fibrinogen-seconds; GLOB, Globulin; HCT, Hematocrit; HCO_3_⁻, Bicarbonate; HGB, Hemoglobin; INR, International Normalized Ratio; K, Potassium; LDH, Lactate Dehydrogenase; LY%, Lymphocyte Percentage; LY#, Lymphocyte Absolute Count; MCH, Mean Corpuscular Hemoglobin; MCHC, Mean Corpuscular Hemoglobin Concentration; MCV, Mean Corpuscular Volume; MO%, Monocyte Percentage; MO#, Monocyte Absolute Count; MPV, Mean Platelet Volume; Mb, Myoglobin; Na, Sodium; NE%, Neutrophil Percentage; NE#, Neutrophil Absolute Count; NT-proBNP, N-terminal pro-B-type Natriuretic Peptide; P, Phosphorus; PCT, Plateletcrit; PDW, Platelet Distribution Width; PLT, Platelet Count; P-LCR, Large Platelet Ratio; Pre-op Cr, Preoperative last Creatinine; Pre-op Height, Preoperative Height; Pre-op Weight, Preoperative Weight; PT, Prothrombin Time; PTA, Prothrombin Time Activity; PT-R, Prothrombin Time Ratio; RBC, Red Blood Cell Count; RDW-CV, Red Cell Distribution Width-Coefficient of Variation; RDW-SD, Red Blood Cell Distribution Width-Standard Deviation; TBIL, Total Bilirubin; TC, Total Cholesterol; TG, Triglycerides; TP, Total Protein; TnI, Troponin I; TT, Thrombin Time; UA, Uric Acid; WBC, White Blood Cell Count.

Table S2. Collinearity diagnostics for the identified independent risk factors.

|  | C_Tac_ (μg/ml) | Pre-op Weight (kg) | CYP3A5(1) | CYP3A5(2) | CYP3A5(3) | RDW-SD (fL) | Mb (ng/mL) | PTA (%) | Na (mmol/L) | Ca (mmol/L) |
| --- | --- | --- | --- | --- | --- | --- | --- | --- | --- | --- |
| C_Tac_ (μg/ml) | 1 | -0.183 | 0.054 | 0.235 | 0.174 | -0.088 | -0.013 | -0.088 | 0.033 | 0.066 |
| Pre-op Weight (kg) | -0.183 | 1 | 0.134 | 0.092 | -0.086 | 0.115 | -0.256 | 0.109 | -0.107 | 0.091 |
| CYP3A5(1) | 0.054 | 0.134 | 1 | 0.285 | 0.358 | 0.061 | -0.006 | 0.16 | -0.052 | -0.006 |
| CYP3A5(2) | 0.235 | 0.092 | 0.285 | 1 | 0.341 | 0.001 | 0.099 | 0.244 | -0.035 | -0.099 |
| CYP3A5(3) | 0.174 | -0.086 | 0.358 | 0.341 | 1 | 0.108 | -0.087 | 0.134 | -0.117 | -0.145 |
| RDW-SD (fL) | -0.088 | 0.115 | 0.061 | 0.001 | 0.108 | 1 | 0.104 | -0.056 | 0.055 | -0.118 |
| Mb (ng/mL) | -0.013 | -0.256 | -0.006 | 0.099 | -0.087 | 0.104 | 1 | -0.162 | 0.104 | 0.112 |
| PTA (%) | -0.088 | 0.109 | 0.16 | 0.244 | 0.134 | -0.056 | -0.162 | 1 | -0.007 | -0.236 |
| Na (mmol/L) | 0.033 | -0.107 | -0.052 | -0.035 | -0.117 | 0.055 | 0.104 | -0.007 | 1 | -0.002 |
| Ca (mmol/L) | 0.066 | 0.091 | -0.006 | -0.099 | -0.145 | -0.118 | 0.112 | -0.236 | -0.002 | 1 |

Note: Ca, serum calcium concentration; C_tac_, Blood concentration of tacrolimus during the first three months; CYP3A5, Cytochrome P450 3A5; Mb, Myoglobin; Na, Sodium; Pre-op Weight, Preoperative Weight; PTA, Prothrombin Time Activity; RDW-SD, Red Cell Distribution Width-Standard Deviation.

Figure S1. The application of the ROC curve. A, Before surgery. B, During long-term medication after surgery.
